# Management of Vasopressor-Induced Acute Limb Ischemia (VIALI) in Septic Shock

**DOI:** 10.7759/cureus.33118

**Published:** 2022-12-30

**Authors:** Noura Attallah, Esraa Hassan, Abbas B Jama, Shikha Jain, Mohamed Ellabban, Renee Gleitz, Sadik Ali, Mool Chand, Nitesh K Jain, Syed Anjum Khan

**Affiliations:** 1 Critical Care Medicine, Mayo Clinic Health System, Mankato, USA; 2 Internal Medicine, MVJ Medical College, Bengaluru, IND; 3 Internal Medicine, Mayo Clinic Health System, Mankato, USA; 4 Surgery and Trauma, Mayo Clinic Health System, Mankato, USA; 5 Hospital Medicine, Mayo Clinic Health System, Mankato, USA

**Keywords:** vasopressor complications, arterial assist pump, icu patients, tissue necrosis, ischemic gangrene, acute limb ischaemia, management of vasopressor-induced acute limb ischemia, vasopressor-induced acute limb ischemia, septic shock, vasopressor

## Abstract

Vasopressors used in critically ill patients with refractory shock poses a serious risk of non-occlusive peripheral limb ischemia leading to tissue necrosis and amputation. Acute limb ischemia is associated with high morbidity and mortality. Evidence-based medical literature is scarce on the prevention and management of vasopressor-induced acute limb ischemia (VIALI). Despite being a well-known and frequent complication of vasopressors, there is no standardized guideline for the prevention and management of vasopressor-induced limb ischemia.

Vasopressors are required for the management of refractory shock which is defined as hypotension not responsive to intravenous fluid resuscitation alone. Distributive shock, which includes septic shock, causes inadequate tissue perfusion in adjunct with vasopressor use and is the most common cause of non-occlusive peripheral limb ischemia. This case study will focus on how early recognition and prompt treatment of VIALI are crucial in minimizing tissue necrosis and preventing amputations.

We present a case of a middle-aged woman who developed distributive shock from sepsis of a urinary source secondary to obstructive uropathy (ureteral calculi). She presented with refractory shock and continued to remain in shock while undergoing emergent rigid cystoscopy with the placement of a ureteral stent. Despite adequate volume resuscitation, she required high doses of vasopressors resulting in peripheral extremity ischemia and necrosis of all her fingers and toes. By promptly initiating mitigation and preventive management strategies, we succeeded in minimizing tissue ischemia and reducing morbidity resulting from iatrogenic vasopressor-induced peripheral non-occlusive ischemia. These strategies include but are not limited to external warming of bilateral lower extremities, nitroglycerin paste application over the entire extremity, arterial assist pump, and low-dose therapeutic anticoagulation. The novel use of the arterial pump in acutely ischemic lower extremities likely helped salvage the toes which appeared to be at high risk of amputation.

## Introduction

Sepsis and septic shock are life-threatening conditions caused by a misaligned immune response to infections which can damage organs and tissues resulting in significant morbidity and mortality, and is a leading cause of death in non-coronary intensive care units [1,2]. Septic shock is defined as hypotension despite adequate fluid resuscitation and is accompanied by organ dysfunction. The urinary tract is the source of 9% to 31% of sepsis cases [3,4]. We present a case report of a 56-year-old woman with septic shock from obstructive nephrolithiasis complicated by vasopressor-induced acute limb ischemia (VIALI).

## Case presentation

A 56-year-old woman presented to an outside emergency room with a fever of 102 F, vomiting, right lower abdominal pain, a heart rate of 142 beats per minute, and blood pressure of 102/66 (mmHg). On examination, she appeared dehydrated, with loss of skin turgor. Her abdominal examination was non-distended with tenderness in the right lower quadrant. Her laboratory workup was significant for leukocytosis, and thrombocytopenia, and was highly suggestive of urinary tract infection (Tables 1, 2).

**Table 1:** Laboratory examinations *E. coli*:  *Escherichia coli*

Laboratory	ICU Admission	Reference Range
Complete Blood Count (CBC)		
Leukocytes	7.8	3.4 -9.6 x 10(9)/L
Neutrophils	2.60	1.56 - 6.45 x 10(9)/L
Hemoglobin	9.7	11.6-15.0 g/dL
Hematocrit	30.6	35.5-44.9%
Platelets	68	150,000-400,000/mm^3^
Blood culture	*E. coli *positive	* E. coli* negative

**Table 2:** Urine analysis

Urine Analysis	ICU Admission	Reference Range
Leukocyte Esterase	Large	Negative
Clarity	Turbid	Clear
Specific Gravity	>1.035	1.001-1.035
Protein	100	Negative
Blood	Large	Negative
White Blood Cells	>100	0-10

**Figure 1:**
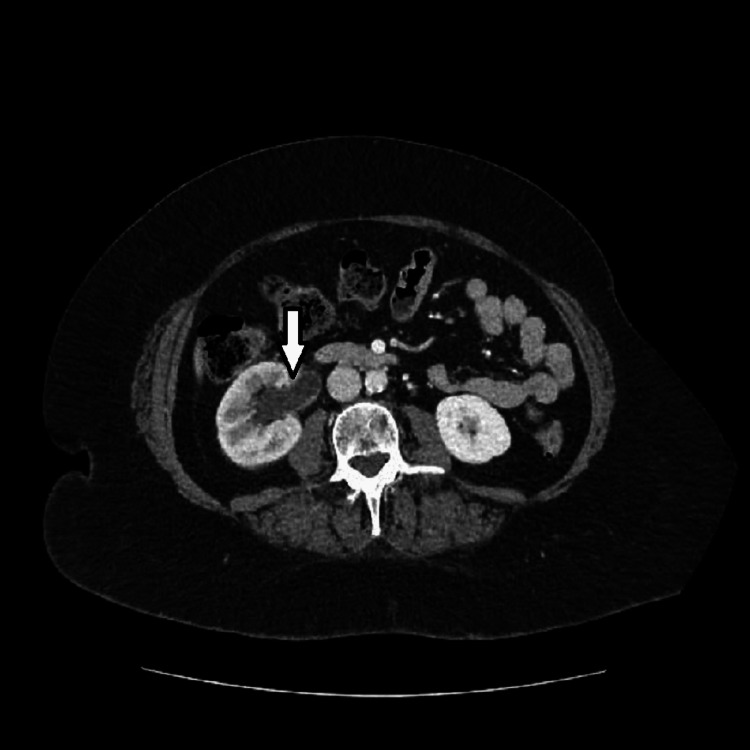
CT scan showing right-sided hydronephrosis

Abdominal-pelvic CT scan showed a right proximal ureter stone measuring 8 x 9 mm causing right-sided hydronephrosis (Figure 1). The patient was initiated on broad-spectrum antibiotics and crystalloid fluid resuscitation.

The patient was transferred to our hospital where she underwent an emergency rigid cystoscopy followed by a right retrograde pyelogram which revealed right-sided hydronephrosis. She underwent a successful right-sided 6 French (F) double-J ureteral stent and indwelling Foley catheter placement. The patient remained hypotensive during the procedure despite fluid resuscitation requiring very high doses of vasopressors which included norepinephrine, epinephrine, vasopressin infusion, and phenylephrine/ephedrine boluses. She required intubation and mechanical ventilation. Following the procedure, she was transferred to the intensive care unit in a hemodynamically unstable condition on mechanical ventilation. She was noted to be hypotensive with a BP of 90/48 (mmHg) and a heart rate of 120 (bpm). She was continued on vasopressor support to target mean arterial pressure (MAP) of greater than 65 (mmHg).

She was initially treated with intravenous ceftriaxone and vancomycin. Metronidazole was added due to suspicion of renal abscess on the CT scan. She was also given stress-dose steroids as she required multiple vasopressors for presumed adrenal insufficiency.

Blood cultures were positive for pan-sensitive *Escherichia coli* within nine hours in all three bottles and subsequently, urine culture was also positive for *E*.* coli*. Hence vancomycin was discontinued.

**Figure 2:**
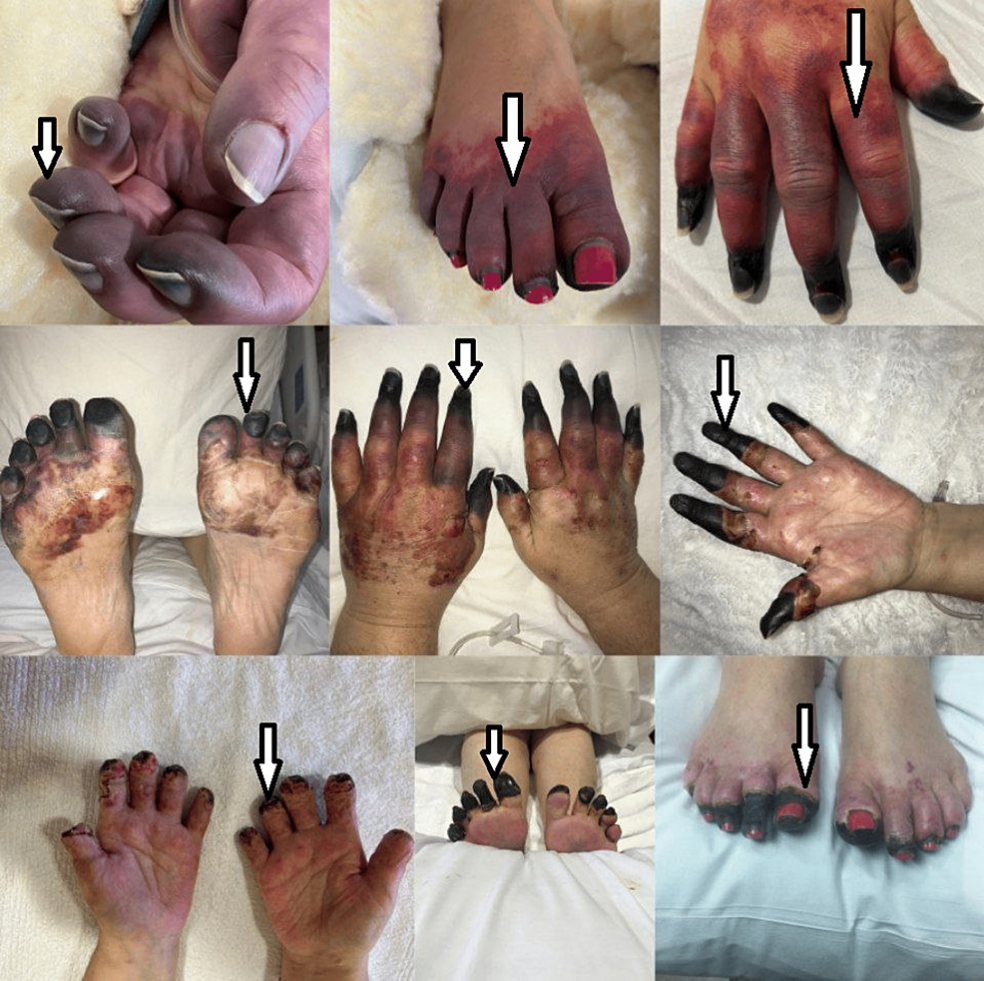
Ischemic changes in both upper and lower extremities

The patient developed significant upper extremity cyanosis with ischemic changes extending from fingertips to the dorsum and palms of her hands, distal to the wrists (Figure 2). Similar cyanosis and ischemic changes were noted in the toes bilaterally (Figure 2).

A dark purple discoloration was noticed which was alarming for necrotic changes to the fingertips. On examination of the lower limb, there was diminished sensation in the distal toes with palpable dorsalis pedis and posterior tibial pulses. Upper extremity doppler ultrasound (US) was obtained which showed bilateral normal arterial flow with no evidence of any thrombosis. Lower extremity doppler US showed bilateral biphasic flow with normal velocities and no thrombosis. Ankle-brachial index (ABI) was normal in both lower limbs. 

A hand and foot wound care consult was obtained. The recommendations included washing in between fingers and toes with normal saline, drying and applying betadine, continuous Rooke vascular boots, applying bear huggers for warming, applying two inches of nitro paste on each affected limb daily at bedtime for six hours followed by wrapping fingers individually with sock-mitts and documenting the skin changes by images. Interventional radiology recommended starting a very low therapeutic dose of heparin to treat any possible micro thrombosis.

ArtAssist Arterial Pump® (ACI Medical, San Marcos, CA, USA) was applied to both the upper and lower extremities. The ArtAssist Pump was discontinued on the upper extremities due to the patient’s discomfort but continued in the lower extremities. All upper extremity bilateral digits had varying degrees of dry gangrene. Bilateral distal interphalangeal joints (DIP) of the upper extremities displayed dry gangrene and frank necrosis. The patient was extubated, weaned off vasopressors gradually, and transferred to the medical ward. Antibiotics were completed and after gangrene demarcation, she was taken to surgery; individual dissection of each digit revealed that the distal phalanx of the index, middle, ring, and little finger were all compromised with full-thickness necrosis without any bleeding tissue. She underwent bilateral revision amputation of the index, middle, ring, and small fingers followed by amputation of the distal aspect of the middle phalanx and the interphalangeal joint of the thumbs. All necrotic tissue was removed with a sagittal saw and rongeur until bleeding tissue was exposed. To ensure adequate soft tissue covering, the bone was significantly debrided. The objective was to promote secondary intention healing. Dorsal and volar soft tissues were lightly reapproximated using a chromic suture. The toes of the lower extremities showed ischemic changes initially but fortunately, perfusion was restored and did not require amputation. On follow-up, the patient is reportedly doing well at home after some rehabilitation. 

## Discussion

Norepinephrine remains the first-line therapy in the treatment of septic shock and is associated with favorable outcomes. However, it carries the risk of end-organ hypoperfusion [5]. In a systematic review that included 153 research studies, eight reported the use of high-dose vasopressors which was defined as more than 0.5 mcg/kg/min with a range of 0.58 to 4 mcg/kg/min for treatment durations of two to 84 hours [6]. Out of these eight studies, only three studies reported limb and digit ischemia or necrosis [6]. Symmetrical peripheral gangrene (SPG) is defined as bilateral peripheral extremities gangrene in the absence of any major vascular occlusion. Distal extremity necrosis is a well-established side effect of vasopressors in critical care patients. In a retrospective study that included 98 ICU patients, 80 patients were exposed to vasoactive drugs, with phenylephrine (55%), norepinephrine (47%), ephedrine (31%), epinephrine (26%), and vasopressin (24%) being the most prevalent [7]. In 70 patients, 40 unilateral and 30 bilateral finger photoplethysmography results were abnormal and five patients ultimately required amputations [7]. Therefore, in clinical settings, the skin should be regularly monitored for any signs of SPG such as pallor, coldness, mottling, cyanosis, and pain in distal limbs during vasopressor use.

High pressure, rapid sequence, intermittent pneumatic calf, and foot compression (HPIPC) devices are considered an alternative therapy in patients with chronic lower limb ischemia who are not candidates for arterial reconstruction surgery [8]. Alvarez et al. reported marked improvement in wound healing, overall pain, and physical activity in patients with chronic peripheral artery disease (PAD) after using HPIPC for four months. Our patient developed progressive ischemia of the digits in the intensive care unit despite the aforementioned supporting measures. After ruling out any thrombosis in the limb vessels, in desperation, we placed an arterial assist device (ART) in the lower extremities to improve vascular flow to the digits [9]. Unfortunately, our patient did not tolerate the placement of the ART device on the upper extremities.

As per the manufacturer, the ArtAssist Pump has been indicated for chronic lower limb ischemia to increase arterial blood flow. It applies a unique compression sequence of 120 mmHg to the foot, ankle, and calf in sequence with comfortable cuffs to circulate blood flow. First, the device compresses the foot and ankle followed by the calf [9]. As a result, the foot, ankle, and calf veins are emptied sequentially, with no effect on the arterial vasculature [9]. Following the emptying of the veins, the pressure gradient between the arterial and venous system is increased which promotes the arterial blood flow down to the toes and ischemic tissues. Because of this mechanism, blood flow to the skin of the feet is enhanced. This sudden increase in blood flow results in a shear force on the vessel endothelium which causes the release of vasodilators (prostacyclin and nitric oxide) [10]. These vasodilators subsequently decrease venous resistance and interrupt arteriolar-venous reflux [9]. Using an HPIPC device early in treating our patient likely resulted in limiting the extent of the ischemia and helped restore perfusion to salvageable tissues in the lower extremities, hence preventing amputation of toes.

## Conclusions

The use of vasopressors in the management of septic shock is vital. In this case report we have described a well-known but infrequently occurring multiple digital ischemias, a serious complication resulting from the use of high-dose vasopressors. It is crucial to remain vigilant for any early signs of limb ischemia and start recommended mitigation therapies to limit the extent of the damage. The main management of VIALI includes decreasing the dosage of vasopressor as tolerated, and other supportive measures as outlined above. Here we highlight the use of an HPIPC device in the management of VIALI. Evidence supports the use of HPIPC devices in treating patients with chronic peripheral arterial disease. However, to our knowledge, HPIPC devices have not been reported to be used in VIALI management to date. We would recommend further research on their use in VIALI.
